# The MnO_2_/GelMA Composite Hydrogels Improve the ROS Microenvironment of Annulus Fibrosus Cells by Promoting the Antioxidant and Autophagy through the SIRT1/NRF2 Pathway

**DOI:** 10.3390/gels10050333

**Published:** 2024-05-15

**Authors:** Bohan Xu, Mingxuan Huang, Jiaying Li, Qingchen Meng, Jie Hu, Qianglong Chen, Hui He, Hao Jiang, Fengxuan Han, Bin Meng, Ting Liang

**Affiliations:** Medical 3D Printing Center, Orthopedic Institute, School of Biology and Basic Medical Sciences, Suzhou Medical College, Soochow University, Suzhou 215000, China; 2030506022@stu.suda.edu.cn (B.X.); 2030506025@stu.suda.edu.cn (M.H.); lijiaying@suda.edu.cn (J.L.); mengqingchen_0406@163.com (Q.M.); hujie_doctor@163.com (J.H.); cql13812441034@163.com (Q.C.); hehui5055@163.com (H.H.); 19979955726@163.com (H.J.); fxhan@suda.edu.cn (F.H.)

**Keywords:** manganese dioxide, reactive oxygen species, antioxidant, autophagy, intervertebral disc degeneration

## Abstract

Intervertebral disc degeneration (IVDD) is a worldwide disease that causes low back pain and reduces quality of life. Biotherapeutic strategies based on tissue engineering alternatives, such as intervertebral disc scaffolds, supplemented by drug-targeted therapy have brought new hope for IVDD. In this study, to explore the role and mechanism of MnO_2_/GelMA composite hydrogels in alleviating IVDD, we prepared composite hydrogels with MnO_2_ and methacrylate gelatin (GelMA) and characterized them using compression testing and transmission electron microscopy (TEM). Annulus fibrosus cells (AFCs) were cultured in the composite hydrogels to verify biocompatibility by live/dead and cytoskeleton staining. Cell viability assays and a reactive oxygen species (ROS) probe were used to analyze the protective effect of the composite hydrogels under oxidative damage. To explore the mechanism of improving the microenvironment, we detected the expression levels of antioxidant and autophagy-related genes and proteins by qPCR and Western blotting. We found that the MnO_2_/GelMA composite hydrogels exhibited excellent biocompatibility and a porous structure, which promoted cell proliferation. The addition of MnO_2_ nanoparticles to GelMA cleared ROS in AFCs and induced the expression of antioxidant and cellular autophagy through the common SIRT1/NRF2 pathway. Therefore, the MnO_2_/GelMA composite hydrogels, which can improve the disc microenvironment through scavenging intracellular ROS and resisting oxidative damage, have great application prospects in the treatment of IVDD.

## 1. Introduction

Low back pain due to intervertebral disc degeneration (IVDD) is a common orthopedic condition which has a serious impact on the quality of life of patients and affects approximately 700 million people worldwide [1,2]. Statistically, low back pain leads to more than 30% of work absenteeism and adds significant healthcare costs [3]. In addition, with the popularity of, and reliance on, the use of electronic devices such as cell phones, tablets, and computers, prolonged periods of faulty posture have led to a trend toward a younger population with degenerative disc disease. Therefore, the treatment of IVDD is of great economic and social significance. Currently, clinical treatments for degenerative disc disease, including medication or physical therapy for symptomatic relief and surgery for restoring the stability of the vertebral structure, may cause re-degeneration or adjacent segment degeneration. At the same time, these can only relieve symptoms, not fundamentally solve the degeneration problem. Biotherapeutic strategies based on tissue engineering alternatives, such as intervertebral disc scaffolds, supplemented by drug-targeted therapy have brought new hope for intervertebral disc degenerative diseases.

The intervertebral disc (IVD) is a fibrocartilaginous tissue connecting the vertebral bodies, and it is mainly composed of the annulus fibrosus (AF), nucleus pulposus (NP), and cartilaginous endplates. Among them, the AF bears compressive and tensile forces around the nucleus pulposus and fixes the nucleus pulposus well, and it plays an essential role in maintaining the structural integrity and biological function of the IVD [4]. During the development of IVDD, due to limited blood supply, self-healing of injuries to the AF is inhibited [5]. The construction of biomaterials that can reproduce AF function is an important strategy for the treatment of IVDD. Biomaterials to replace the AF microenvironment could be roughly divided into natural materials (including proteins, polysaccharides, and peptides) and synthetic materials (including a variety of physical and chemical crosslinked polymers), which have good biocompatibility and mechanical properties, respectively [6]. However, natural biomaterials (such as alginate and fibrinogen hydrogels) have often been found to have poor biomechanical properties and have thus been unable to meet the requirements of IVD pressure loading, while synthetic biomaterials (such as glass/sodium and polyvinyl alcohol hydrogels) have been found to have poor cell adhesion or be cytotoxic [7]. Therefore, it is still necessary to explore materials that meet both mechanical load and biocompatibility requirements for IVDD. Methacrylated gelatin anhydride (GelMA), as a photosensitive biological hydrogel material, has good biocompatibility and can crosslink quickly under visible or ultraviolet light. Scaffolds prepared with GelMA as the substrate can form three-dimensional structures suitable for cell proliferation and differentiation with a certain strength and have been widely used in bone tissue engineering [8].

Although current bionic intervertebral disc scaffolds have made significant progress in regard to their preparation processes and materials, they still have not achieved the therapeutic effect of improving the microenvironment and promoting the repair and regeneration of degenerated intervertebral discs. The microenvironmental changes in degenerated discs are complex, with the presence of multiple inflammatory factors, active proteases, elevated reactive oxygen species (ROS) [9,10,11], decreased pH, and reduced glucose/oxygen [12]. Among them, ROS is an important factor in the mechanism of disc degeneration, and these species mainly include hydrogen peroxide (H_2_O_2_), superoxide anions (O_2_^−^), and hydroxyl radicals (HO^−^). Accumulation of ROS not only leads to the destruction of the extracellular matrix (ECM) with death and apoptosis of the annulus fibrosus cells, but also the activation of inflammatory pathways [13,14]. In addition, the overproduction of ROS significantly down-regulates the expression of type II collagen and aggrecan. It induces the overexpression of catabolic factors, such as matrix metalloproteinase [15], which ultimately leads to disruption of the balance of synthetic catabolism in the ECM and accelerates the progression of IVDD.

Several small molecule antioxidants, such as melatonin and N-acetylcysteine, have been reported to scavenge ROS and inhibit oxidative stress [16,17]. However, the therapeutic efficacy of small molecule antioxidants is usually limited by their low efficiency in scavenging ROS, and traditional administration often fails to effectively aggregate at the lesion site. They are also unable to solve the problem of insufficient oxygen supply in the degenerative microenvironment [18]. Therefore, the development of novel therapeutic regimens with efficient and targeted ROS scavenging is essential for IVDD.

Manganese dioxide (MnO_2_) nanoparticles with a hollow structure have been found to efficiently scour ROS and promote tissue repair. In addition, they can provide oxygen supply in response [19,20]. Previous reports have confirmed that hollow MnO_2_ nanoparticles can excellently regulate the level of ROS and increase the expression of antioxidant-related proteins such as SOD2 in the acidic microenvironment of bone defects [21]. Moreover, hollow MnO_2_ nanoparticles can also be used as good drug carriers to carry therapeutic drugs and achieve pH/H_2_O_2_ responsive drug release [22]. Thus, MnO_2_ has potential application in IVDD.

In this study, we developed a composite hydrogel containing hollow MnO_2_ nanoparticles, which could directly scavenge ROS in the degenerative microenvironment of IVD, and improve the expression of antioxidant genes through the SIRT1/NRF2 pathway to enhance the intrinsic cellular resistance to oxidative stress, thereby achieving a therapeutic effect on IVDD. In addition, the biocompatibility and mechanical properties of the composites were evaluated, and the underlying mechanism of these composite hydrogels on IVD cells was explored in this study.

## 2. Results and Discussion

### 2.1. Characterizations of MnO_2_ Nanoparticles and Composite Hydrogels

**Figure 1:**
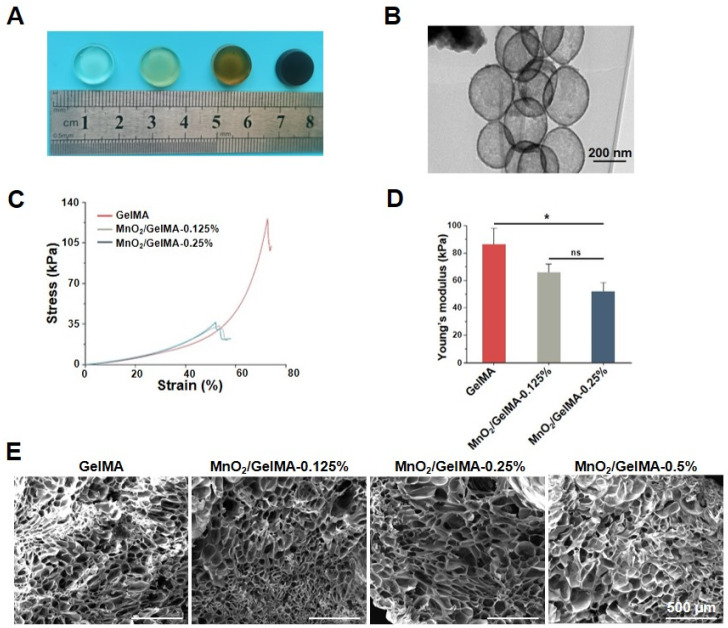
Characterizations of MnO_2_/GelMA composite hydrogels. The gross morphology of the composite hydrogels is displayed (**A**). The morphology of MnO_2_ (**B**) was observed by using transmission electron microscopy (TEM). Compressive stress–strain curves (**C**) and Young’s modulus (**D**) of the GelMA composite hydrogels with different MnO_2_ concentrations were analyzed via compression test, and the morphology of the MnO_2_/GelMA composite hydrogels (**E**) was observed using scanning electron microscopy (SEM). Data are presented as mean ± SD (*n* = 3). ns *p* > 0.05; * *p* < 0.05, versus hydrogels without MnO_2_ (GelMA) (**D**). Representative images are shown. Scale of TEM images = 200 nm (**B**) and scale of SEM images = 500 μm (**E**).

MnO_2_ nanoparticles with hollow structures were prepared by core-shell synthesis. Then, they were added to GelMA solution to produce composite hydrogels, which contained nanoparticles with different concentration gradients (Figure 1A). After the synthesis of hollow MnO_2_ nanoparticles, their morphology was observed using transmission electron microscopy (TEM) (Figure 1B). The hollow MnO_2_ nanoparticles were regular spherical, with diameters mainly distributed from 250 to 300 nm, and an average diameter of 268 nm. The mechanical properties of the MnO_2_/GelMA composite hydrogels were analyzed by compression test. The results revealed that both the compression strength and elastic modulus of the composite hydrogels decreased with the addition of MnO_2_ concentration (Figure 1C,D). Scanning electron microscopy (SEM) showed that the pore size of composite hydrogels with different nanoparticle concentrations did not change significantly, and the morphology was regular (Figure 1E).

### 2.2. Biocompatibility of MnO_2_/GelMA Composite Hydrogels

**Figure 2:**
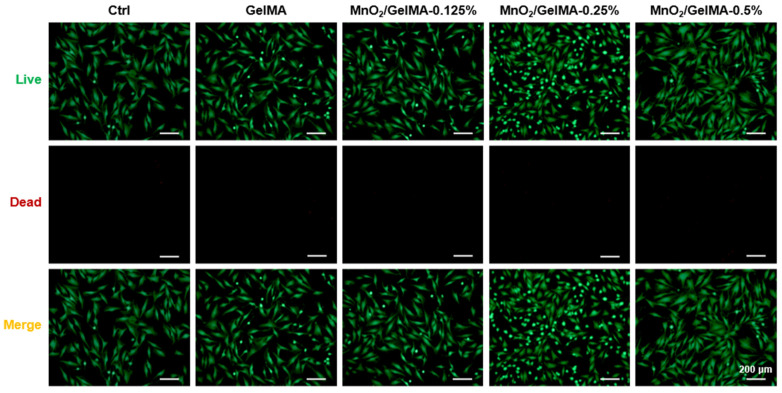
Live/dead staining images of AFCs were demonstrated to show live (green) or dead (red) cells in MnO_2_/GelMA composite hydrogels, and the color merged into yellow where the two survival conditions overlap. Representative images are shown. Scale = 200 μm.

**Figure 3:**
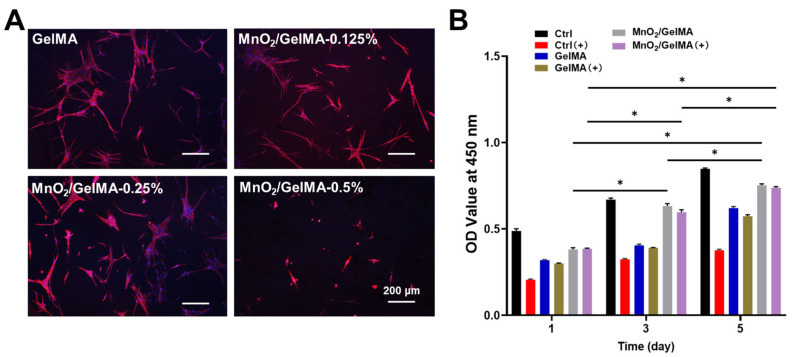
The nucleus and cytoskeleton of AFCs seeded on different hydrogels were stained with DAPI (blue) and phalloidin (red) (**A**). Cell proliferation of AFCs was analyzed via CCK-8 assay on days 1, 3, and 5, with or without H_2_O_2_, which is represented by (+) (**B**). Data are presented as mean ± SD (*n* = 3). * *p* < 0.05, versus the group treated with the same condition on different days (**B**). Representative images are shown. Scale = 200 μm (**A**).

Composite hydrogels with different MnO_2_ concentrations were utilized to test their biocompatibility. Live/dead staining results showed that AFCs grew well in composite hydrogels with concentrations lower than 0.5%, similar to the control group, while there was cell swelling at 0.5% concentration (Figure 2). Fluorescence staining of the cytoskeleton (Figure 3A) revealed that with MnO_2_ concentrations of 0.125% and 0.25%, the AFCs adhered and spread well on the composite hydrogels, suggesting excellent biocompatibility. However, when the concentration was increased to 0.5%, AFCs underwent shrinkage denaturation and lost their normal spreading appearance, indicating that the composite hydrogels with a higher MnO_2_ concentration (0.5%) were less biocompatible. The results of the CCK-8 assay with or without H_2_O_2_ suggested that the oxidative damage induced by H_2_O_2_ significantly inhibited the proliferation of AFCs, while MnO_2_/GelMA composite hydrogels could restore the proliferation (Figure 3B). Based on these results, we chose the composite hydrogels with an MnO_2_ concentration of 0.25% for subsequent experiments.

### 2.3. Scavenging Effect of Composite Hydrogels on Intracellular ROS

**Figure 4:**
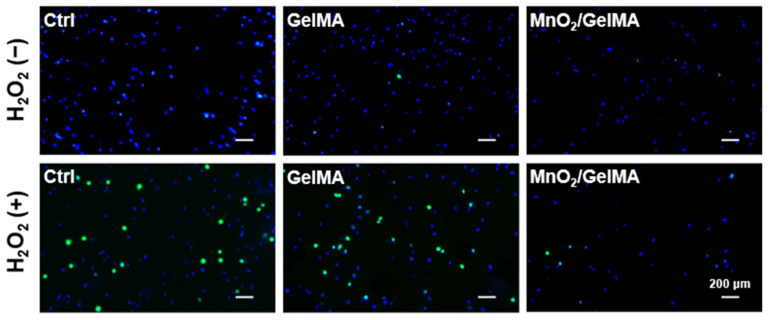
ROS-positive AFCs in various microenvironments labeled with DCFH-DA (green) and cell nuclei were stained with DAPI (blue). Representative images are shown. Scale = 200 μm.

To simulate the microenvironmental characteristics of oxidative stress in degenerative intervertebral discs, we treated AFCs with H_2_O_2_. After that, the ROS levels of AFCs exposed to H_2_O_2_ were detected using the ROS fluorescent probe DCFH-DA. The results revealed that H_2_O_2_ significantly increased the intracellular ROS levels of AFCs in the control and GelMA groups. In contrast, it had no significant impact on the intracellular ROS level of AFCs in the MnO_2_/GelMA composite hydrogels (Figure 4), which indicated that the MnO_2_/GelMA composite hydrogels had intracellular antioxidant damage effects.

### 2.4. Antioxidant Effects of MnO_2_/GelMA Composite Hydrogels on AFCs

**Figure 5:**
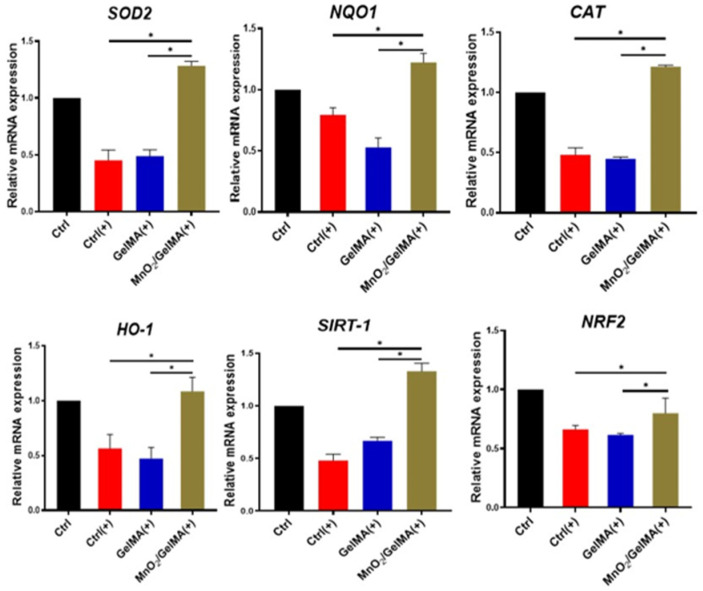
The expression of antioxidant genes in AFCs under oxidative stress analyzed by qPCR. Data are presented as mean ± SD (*n* = 3). * *p* < 0.05.

**Figure 6:**
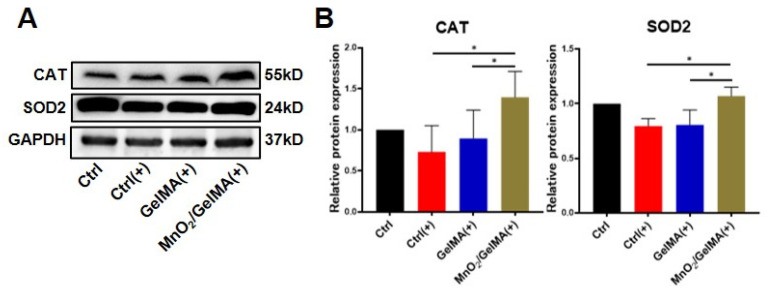
Antioxidant protein expression levels of AFCs in different microenvironments determined by Western blotting (**A**), and quantitative analysis of resulting images (**B**). Data are presented as mean ± SD (*n* = 3). * *p* < 0.05.

To further elucidate the mechanism of composite hydrogels-mediated cellular antioxidant activity, we examined the expression levels of antioxidant genes in AFCs, including superoxide dismutase 2 (*SOD2*), catalase (*CAT*), NAD(P)H: quinone oxidoreductase 1 (*NQO1*), heme oxygenase-1 (*HO-1*), and their upstream regulatory genes, silent mating-type information regulation 2 homolog 1 (*SIRT1*), and nuclear factor erythroid 2-related factor 2 (*NRF2*). The results (Figure 5) showed that compared with AFCs without any treatment, the expression of the antioxidant genes *SIRT1*, *NRF2*, *SOD2*, *CAT*, *NQO1,* and *HO-1* was significantly down-regulated in the control group and GelMA group exposed to H_2_O_2_, suggesting that the antioxidant damage capacity of the AFCs was reduced. However, the expression of antioxidant genes in AFCs inoculated with MnO_2_/GelMA composite hydrogels treated with H_2_O_2_ was restored to the normal level and increased compared with the tissue culture plate control or GelMA group with H_2_O_2_ treatment, which indicated that the MnO_2_/GelMA composite hydrogels had the function of inducing the antioxidant gene expression of AFCs under the state of oxidative stress. Results of Western blotting similarly also indicated that the disc microenvironment constructed by composite hydrogels can enhance the intrinsic ability of AFCs to resist oxidative damage by promoting the expression of antioxidant proteins SOD2 and CAT (Figure 6A,B). These results suggested that MnO_2_/GelMA composite hydrogels could directly scavenge ROS produced in AFCs and resist oxidative damage by up-regulating expression levels of antioxidant genes and promoting the synthesis of antioxidant protein. In summary, MnO_2_/GelMA composite hydrogels not only directly scavenge ROS produced in AFCs, but also contribute to defense against oxidative damage by modulating the expression or synthesis levels of antioxidant-related genes and proteins.

### 2.5. Effects of Composite Hydrogels in Promoting Cellular Autophagy

**Figure 7:**
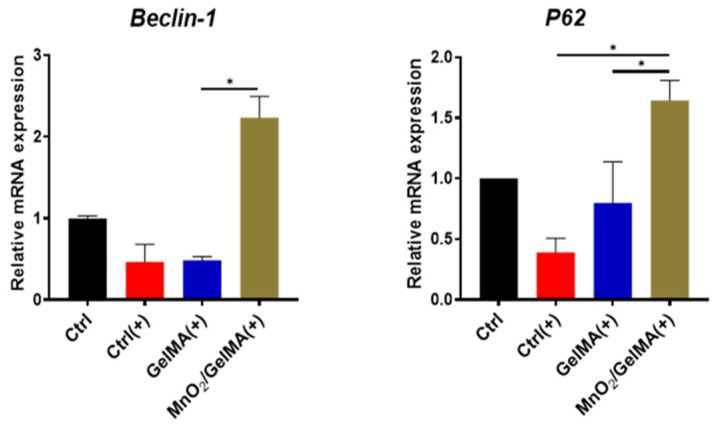
AFCs were treated with H_2_O_2_ in GelMA and MnO_2_/GelMA hydrogels. The expression of autophagy-related genes (*Beclin-1* and *P62*) was quantified. Data are presented as mean ± SD (*n* = 3). * *p* < 0.05.

To investigate the effect of composite hydrogels on autophagy-related antioxidant damage in AFCs, we examined the expression of autophagy-related genes *Beclin-1* and *P62*. The results revealed that the expression of *Beclin-1* and *P62* was down-regulated in AFCs after H_2_O_2_ treatment, while that of the MnO_2_/GelMA group was significantly up-regulated (Figure 7), suggesting that the MnO_2_/GelMA composite hydrogels may be involved in the process of cytoprotection and antioxidant damage by up-regulating the expression of autophagy-related genes.

### 2.6. Discussion of Findings

**Figure 8:**
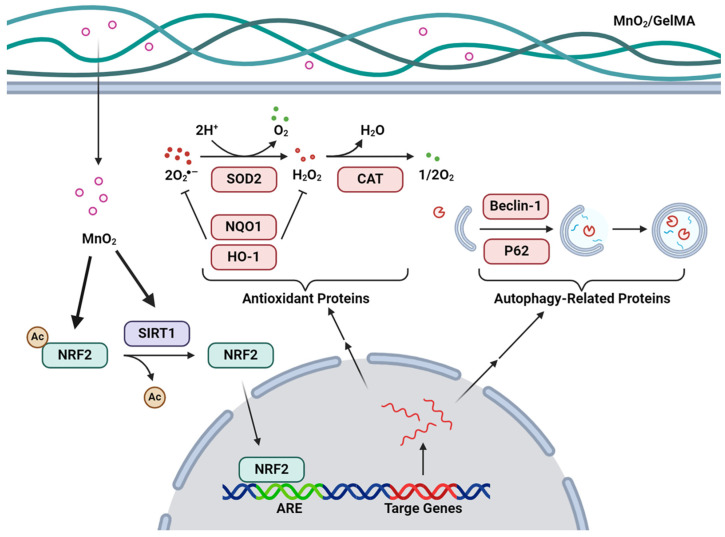
Mechanism of MnO_2_/GelMA composite hydrogels in improving the microenvironment of AFCs. After MnO_2_ in the composite hydrogels enters AFCs, NRF2 and SIRT1 are up-regulated, and SIRT1 activates NRF2 through deacetylation. After activation, NRF2 enters the nucleus and binds to the antioxidant response element (ARE), inducing the expression of antioxidant and autophagy-related genes and proteins. SOD2 and CAT catalyze the conversion of ROS into stable molecules such as O_2_, while NQO1 and HO-1 can also consume ROS to achieve antioxidant function. P62 and Beclin-1 participate in the formation of autophagosomes and promote the clearance of oxidation-damaged structures.

In this study, we engineered MnO_2_/GelMA composite hydrogels with good biocompatibility and efficient scavenging of microenvironmental ROS to promote IVDD repair. The composite hydrogels can significantly reduce ROS in AFCs and release O_2_ to improve the acidic and hypoxic microenvironment. The composite hydrogels can also ameliorate the intrinsic antioxidant capacity of the cells by up-regulating the expression of genes related to cell antioxidant and autophagy, which can help AFCs better cope with the changes in the microenvironment associated with intervertebral disc degeneration, and thus delay and cure or relieve intervertebral disc degeneration (Figure 8).

The intervertebral disc, as the largest avascular tissue in the body, transmits nutrients through the vertebral endplates, mainly by diffusion [23]. However, degenerated intervertebral disc tissues have reduced permeability and accumulation of metabolic substances, resulting in a poor microenvironment of low pH and high ROS. In our experiments, we observed that H_2_O_2_ induced an increase in ROS levels in AFCs. The accumulation of ROS can lead to cell cycle arrest and apoptosis, affect the balance of anabolism and catabolism of the extracellular matrix, and then accelerate the process of intervertebral disc degeneration, thus forming a vicious cycle [24].

Therefore, it is significant to regulate ROS levels to inhibit oxidative stress for the treatment of disc degeneration. Some molecules such as dimethyl fumarate [25], N-acetylcysteine [26], and nanoparticles such as nano-enzymes [27], have been used to eliminate intracellular ROS and play essential roles in maintaining mitochondrial homeostasis and inhibiting cellular senescence. In this study, we built a ROS-resistant material consisting of MnO_2_ and GelMA hydrogels. MnO_2_ nanoparticles can not only directly remove H_2_O_2_ from the microenvironment, but also decompose it into H_2_O and O_2_. In addition, MnO_2_ relatively easily undergoes a reversible redox reaction in an acidic environment and produces Mn_3_O_4_, which in turn can be used as a catalyst to degrade ROS, such as hydroxyl, to less reactive species [28]. This is beneficial to alleviate the hypoxia of degenerative tissues and protect the annulus fibrosus and nucleus pulposus tissues from inflammation-induced oxidative damage. As a biocompatible and photo-crosslinkable hydrogel, GelMA is quite popular in tissue engineering and is a suitable carrier loaded with nanoparticles to continuously remove ROS to promote the repair of degenerative intervertebral discs.

In addition to the ability to scavenge ROS, we also found that the MnO_2_/GelMA composite hydrogels up-regulated the expression of antioxidant genes *SITR-1*, *NRF2*, *SOD2, CAT*, *NQO1*, and *HO-1*, which could promote a reduction in ROS levels and protect AFCs from oxidative damage. SIRT1 is a widely expressed NAD-dependent deacetylase, which is closely related to preventing oxidative stress and inflammation [29]. NRF2, a transcription factor of the basic leucine (bZIP) family, can be activated by SIRT1 deacetylation [30]. When ROS levels are elevated and activate the SIRT1/NRF2 pathway, NRF2 promotes the expression of downstream antioxidant proteins such as SOD2, CAT, NQO1, and HO-1, which play a role in scavenging ROS and releasing O_2_ [31]. Many studies have shown that the expression of antioxidant genes and proteins can protect intervertebral disc cells and improve disc degeneration. Resveratrol can inhibit AFCs apoptosis in the inflammatory environment by up-regulating SOD2 expression [32]. Fucoidan-loaded nanofibrous scaffolds improve degenerative disc inflammation and promote annulus fibrosus repair by enhancing the expression of antioxidant proteins such as NRF2 and HO-1 [33]. Therefore, activation of intracellular antioxidant function is an important avenue for the prevention and treatment of IVDD, and our study suggests the potential value of MnO_2_/GelMA composite hydrogels in this process.

Autophagy, as an important mechanism to remove damaged structures in cells, is particularly important in reducing oxidative damage and ROS levels to maintain cellular homeostasis. More and more studies have focused on the important role of autophagy in intervertebral disc degeneration [16,34,35]. As mentioned above, in addition to promoting the expression of antioxidant genes, NRF2 can also induce the transcription of genes, including the autophagy-related protein p62 [36]. P62 acts as a molecular adapter between the autophagy mechanism and its substrate to selectively degrade damaged structures marked by ubiquitination, thereby reducing oxidative damage [37,38]. Another autophagy-related protein, Beclin-1 (also known as Atg6), is the backbone of the core complex required for autophagosome formation [39]. Up-regulation of the expression of both autophagy-related genes *P62* and *Beclin-1* is an important marker of autophagy activation [40]. Our study found that the MnO_2_/GelMA composite hydrogels promoted the expression of the autophagy-related genes *P62* and *Beclin-1*, suggesting that part of their anti-oxidative damage effect may be achieved through the autophagy mechanism. Studies have shown that IL-1β protects AFCs from apoptosis by up-regulating autophagy induced by serum deprivation [41]. Meanwhile, BM-MSC-derived exosomes inhibit IL-1β-induced AFCs inflammation and apoptosis by inhibiting autophagy [42]. However, a study by Ni et al. found that TGF-β1 could promote AFCs survival under ROS by inhibiting autophagy [43], which is not consistent with the protective effect of autophagy in our study. It suggests the two-sided nature of autophagy function, and the role of autophagy in AFCs under ROS needs to be explored by further studies.

## 3. Conclusions

The MnO_2_/GelMA composite hydrogels in this study have excellent biocompatibility and porous structure and promote cell proliferation. Adding MnO_2_ nanoparticles also enhances the antioxidant property of AFCs and induces cellular autophagy through the common SIRT1/NRF2 pathway, which can prevent intracellular oxidative damage under oxidative stress. In conclusion, the composite hydrogels could improve the disc microenvironment by clearing intracellular ROS and promoting antioxidant and autophagy activity through the SIRT1/NRF2 pathway, thus showing potential in the treatment of disc degeneration. In addition, nanostructured materials based on microenvironment improvement also provide new possibilities for the exploration of bionic intervertebral disc scaffolds.

## 4. Materials and Methods

### 4.1. Preparation of MnO_2_ Nanoparticles

Ethanol (14 mL), ammonia (500 μL), and deionized water (2 mL) were mixed in a flask at a constant temperature of 45 °C with continuous stirring for 5 min. Subsequently, 500 μL of tetraethyl orthosilicate (TEOS) was added dropwise. After completing the addition, the mixture was stirred for 3 h, washed with ethanol and deionized water, and centrifuged to obtain mesoporous SiO_2_ nanoparticles. The nanoparticles were dispersed in water and then 20 mL of aqueous potassium permanganate solution was added dropwise under sonication conditions and continuously sonicated for 1 h. The mixture was then stirred for 12 h and centrifuged, and the supernatant was discarded to obtain SiO_2_@MnO_2_ nanoparticles. Then, these nanoparticles were scattered into sodium for etching over 12 h. Finally, the MnO_2_ nanoparticles were obtained.

### 4.2. Preparation of MnO_2_/GelMA Composite Hydrogels

Under light-avoiding conditions, 0.25% *w/v* photoinitiator lithium phenyl-2,4,6-trimethylbenzoylphosphinate (LAP) and 5% *w/v* GelMA were added to phosphate buffered saline (PBS) solution and dissolved by heating a 55 °C water bath for 20 min. The prepared GelMA solution was decontaminated using a 0.22 μL sterile needle filter on an ultra-clean bench under light protection, then sealed and stored in a refrigerator at 4 °C under light protection.

MnO_2_ nanoparticles were sterilized by adding 75% ethanol and sealed. Different concentrations of MnO_2_ were added to 1 mL of 10% GelMA solution, respectively, and then irradiated using a blue light (wavelength of 405 nm) for 3 min to obtain composite hydrogels.

### 4.3. Morphology of MnO_2_ Nanoparticles and Composite Hydrogels

MnO_2_ nanoparticles were detected by transmission electron microscopy (TECNAI G2 F20, FEI, Hillsboro, OR, USA). The cross-sectional morphology of the MnO_2_/GelMA composite hydrogels was captured using scanning electron microscopy (SEM, Quanta 250, Hillsboro, OR, USA).

### 4.4. Cell Viability and Proliferation Measurements

AFCs were inoculated in 24-well plates at a density of 4 × 10^3^ per well. After 12 h, different composite hydrogels were deposited in the supernumerary cavity, and the culture plate in a simple state was used as a control group. After that, the AFCs proliferation ability was detected by CCK-8 assay.

### 4.5. Cytoskeleton Staining

To explore the biocompatibility of the MnO_2_/GelMA composite hydrogels, AFCs were seeded in GelMA, MnO_2_/GelMA-0.125%, MnO_2_/GelMA-0.25%, and MnO_2_/GelMA-0.5% composite hydrogels, respectively, and the density of cells in each well was made up to be 5 × 10^4^. GelMA tissue culture plates were used as the control. After 48 h of culture, the AFCs were fixed using a 4% formaldehyde solution dissolved in PBS, and after 10 min of fixation, the residual formaldehyde was removed with PBS, then permeabilized with 0.5% Triton X-100 (Sigma-Aldrich, Burlington, MA, USA) solution for 5 min. Subsequently, 3% bovine serum albumin solution was added to each group of the culture plate, and then the samples were treated at 4 °C for 12 h protected from light. After that, the AFCs were co-incubated with phalloidi, and stained with 4′,6-diamidino-2-phenylindole (DAPI). The morphology of the cytoskeleton was observed, and images were captured under an inverted fluorescence microscope (EVOS, Tampa, FI, USA).

### 4.6. Mechanical Properties of Composite Hydrogels

The prepared samples (height, 5 mm; diameter, 4 mm) were put on a platform. Subsequently, the compressive strength was tested at a speed of 1 mm/min. Then, the stress–strain curves and the strength were obtained.

### 4.7. Detection of Intracellular ROS

The suspension of annulus fibrosus cells was added to GelMA hydrogel and MnO_2_/GelMA composite hydrogel, and the cells were treated with 10 × 10^−6^ mol H_2_O_2_ for 24 h, during which they grew uniformly and at a suitable density in the hydrogel (at a density of 1 × 10^4^ per well). The group without hydrogel and the simultaneous culture plates without H_2_O_2_ were set as the control group. Then, 2,7-dichlorofluorescein diacetate was added to each group. Fluorescence imaging was carried out with an inverted fluorescence microscope (EVOS, Tampa, FI, USA).

### 4.8. Quantitative Real-Time Polymerase Chain Reaction (qPCR)

Rat AFCs were seeded into different composite hydrogels, and AFCs cultured in 6-well plates were used as controls, grouped as follows: Ctrl, Ctrl (+), GelMA (+), and MnO_2_/GelMA (+), in which + indicates treatment with H_2_O_2_. After each group was cultured for a certain period, the cells were lysed by adding Trizol lysis solution (Invitrogen, Carlsbad, CA, USA) to obtain cellular RNA. Then, the RNA concentration was measured by NanoDrop (Thermo Fisher Scientific, MA, USA). The amount of substrate required for amplification was calculated from the concentration of RNA, then the RNA was reverse transcribed into cDNA for each sample by a 5XAll-OneRTMasterMix (abm, Vancouver, Canada). The reverse transcription system is shown in Table 1. The obtained cDNAs were configured with the qPCR reaction system. Finally, antioxidant genes (*SIRT-1*, *NIRF2*, *SOD2*, *CAT*, *NQO1*, *HO-1*) and cellular autophagy-related genes (*Beclin-1, P62*) were quantified using real-time PCR. Primer sequences are listed in Table 2.

### 4.9. Western Blotting Analysis

The AFCs were taken on ice, washed three times with PBS, and lysed with RIPA lysis solution (Beyotime, Shanghai, China). The cells at the bottom of the wells were scraped to one side of the well plate using a spatula, collected and placed on ice for 15 min, and shaken every 5 min. After that, the protein concentration of the supernatant was analyzed using a BCA Protein Assay Kit (Beyotime, Shanghai, China).

Proteins were quickly denatured in a metal heater, then underwent electrophoresis, and were transferred to nitrocellulose membranes. The membranes were incubated with a special blocking solution for 1 h at room temperature, mixed with primary antibody, and incubated at 4 °C for 12 h. After that, the membranes were rewarmed for 20 min, and incubated with anti-rabbit or anti-mouse fluorescent secondary antibody for 1 h on a shaker. Proteins were measured by an Odyssey infrared imaging system. The density of the immunoblot signal was quantified by ImageJ (Version 1.54i).

### 4.10. Statistical Analysis

The results were expressed as mean ± SD. One-way analysis of variance (ANOVA) and Tukey’s multiple comparisons were used to assess differences between groups. The analysis was conducted with GraphPad Prism 9.0.0 (USA).

## Figures and Tables

**Table 1:** Reverse transcription system.

Reagent	Volume
Oligo (dT)18 Primer	1 μL
RNA	1/X *
5X All-in-one RT MasterMix	4 μL
Nuclease-free H_2_O	15-1/X

* X is the RNA concentration measured.

**Table 2:** PCR primer sequences.

Target Genes *	Forward Primer Sequence (5′–3′)	Reverse Primer Sequence (5′–3′)
*CAT*	GGCCTGACTGACGCGATTGC	CTGCTCCTTCCACTGCTTCATCTG
*SOD2*	GCTGGAGGCTATCAAGCGTGAC	TTAGAGCAGGCGGCAATCTGTAAG
*SIRT1*	GCTCGCCTTGCTGTGGACTTC	GTGACACAGAGATGGCTGGAACTG
*HO-1*	ACAGACAGAGTTTCTTCGCC	ATAAATTCCCACTGCCACGG
*NRF2*	GCAGGCTGAGACTACCACTG	CTGGCATCATCCGTGGAGAG
*NQO1*	GAAGAGCACTGATCGTACTGGC	GGATACTGAAAGTTCGCAGGG
*Beclin-1*	GGGTCTAAGGCGTCCAGCAG	GACACCATCCTGGCGAGTTT
*P62*	TATTACAGCCAGAGTCAAGG	CTACATACAGAAGCCAGAATG
*GAPDH*	GACCTGACCTGCCGTCTA	AGGAGTGGGTGTCGCTGT

* *CAT*: catalase; *SOD2*: superoxide dismutase 2; *SIRT1*: silent information regulator type 1; *HO-1*: heme oxygenase 1; *NRF2*: nuclear factor erythroid 2-related factor 2; *NQO1*: NAD(P)H Quinone Dehydrogenase 1; *Beclin-1*: Beclin 1; *P62*: sequestosome 1.

## Data Availability

The data that support the findings of this study are available from the corresponding author upon reasonable request.
